# B cell‐activating factors in autoimmune pulmonary alveolar proteinosis

**DOI:** 10.1186/s13023-021-01755-y

**Published:** 2021-03-02

**Authors:** Masaki Hirose, Toru Arai, Chikatoshi Sugimoto, Takayuki Takimoto, Reiko Sugawara, Shojiro Minomo, Sayoko Shintani, Naoko Takeuchi, Kanako Katayama, Yasushi Inoue, Tomoko Kagawa, Takahiko Kasai, Masanori Akira, Yoshikazu Inoue

**Affiliations:** 1 Clinical Research Center, National Hospital Organization Kinki-Chuo Chest Medical Center, 1180 Nagasone-Cho, Kita-Ku, Sakai City, Osaka 591-8555 Japan; 2 Department of Internal Medicine, National Hospital Organization Kinki-Chuo Chest Medical Center, 1180 Nagasone-Cho, Kita-Ku, Sakai City, Osaka 591-8555 Japan; 3 Department of Pathology, National Hospital Organization Kinki-Chuo Chest Medical Center, 1180 Nagasone-Cho, Kita-Ku, Sakai City, Osaka 591-8555 Japan; 4 Department of Radiology, National Hospital Organization Kinki-Chuo Chest Medical Center, 1180 Nagasone-Cho, Kita-Ku, Sakai City, Osaka 591-8555 Japan

**Keywords:** Autoimmune pulmonary alveolar proteinosis, B cell‐activating factor, Biomarker

## Abstract

**Background:**

Autoimmune pulmonary alveolar proteinosis (APAP) results from the suppression of granulocyte-macrophage colony-stimulating factor (GM-CSF) signaling by a neutralizing autoantibody against GM-CSF. B cell-activating factor (BAFF) and a proliferation-inducing ligand (APRIL) are involved in immunoglobulin G production and are overproduced in various autoimmune disorders. We hypothesized that BAFF and/or APRIL levels would be elevated in serum and bronchoalveolar lavage fluid (BALF) and serum and BALF levels of BAFF and APRIL respond to the treatments (whole lung lavage (WLL) or inhalation of recombinant human granulocyte-macrophage colony-stimulating factor (GM-CSF)) in patients with APAP.

**Subjects and methods:**

BAFF and APRIL levels in serum and BALF from 110 patients with APAP were measured at baseline and during and after treatment, using an enzyme-linked immunosorbent assay kit. We enrolled 34 healthy volunteers as serum cytokine controls, and 13 disease controls for BALF. Associations of BAFF and APRIL levels with clinical measures were assessed to clarify their clinical roles.

**Results:**

In patients with APAP, serum BAFF and APRIL levels were significantly increased relative to healthy volunteers (p < 0.0001 and p < 0.05, respectively), and BALF BAFF and APRIL levels were significantly increased versus disease controls (p < 0.0001 and p < 0.01, respectively). Serum BAFF levels (but not APRIL levels) were significantly correlated with Krebs von den Lungen-6 (KL-6), surfactant protein (SP)-D, SP-A, and lactate dehydrogenase (p < 0.0001). There was no significant correlation between serum BAFF or APRIL levels and anti-GM-CSF autoantibody. BAFF and APRIL were negatively correlated with single-breath diffusion capacity for carbon monoxide (DLco) (p = 0.004) and forced vital capacity (p = 0.04), respectively. BAFF (but not APRIL) in BALF was negatively correlated with vital capacity (p = 0.04) and DLco (p = 0.006). There were significant correlations between disease severity and BAFF levels in serum (p = 0.04) and BALF (p = 0.007). Serum levels of anti-GM-CSF autoantibody, BAFF, and APRIL were not significantly affected by WLL or inhalation of recombinant human GM-CSF.

**Conclusions:**

BAFF and APRIL levels of sera and BALF in APAP were significantly increased compared with healthy volunteer and disease control, and the BAFF and APRIL pathway might have important specific roles in pathogenesis of APAP. Our data suggest a new perspective of future treatment for APAP.

## Introduction

Pulmonary alveolar proteinosis (PAP) is a rare lung disease with the low incidence and prevalence [1, 2]. Dysfunction of alveolar macrophages induced by a neutralizing autoantibody against granulocyte-macrophage colony-stimulating factor (GM-CSF) is a pathogenic factor of autoimmune PAP (APAP) [2–5]. However, no correlation has been observed between the disease severity score (DSS) and levels of anti-GM-CSF autoantibody in the serum [1]. The trigger and mechanism of the overproduction of anti-GM-CSF autoantibody are not currently known.

Whole lung lavage (WLL) is the gold standard therapy for APAP [6–8]. In addition, treatment with inhaled recombinant human GM-CSF (rhGM-CSF) has been conducted in clinical trials [9–11]. The recurrence rate of APAP after WLL is unclear, although one report found that 70 % of patients followed up for more than three years remained free of recurrent PAP manifestations [6]. However, another study reported that 71 % of patients receiving treatment with rhGM-CSF had received WLL previously [7]. The recurrence rate of APAP after treatment with rhGM-CSF is also unclear, although it has been reported that symptoms recurred in five out of 12 patients after a mean of 6.3 months [8]. Another study reported that, during 30 months of observation following GM-CSF inhalation therapy, 12 of 35 patients required additional treatment [12]. Therefore, the development of treatments that are more effective and less invasive is desirable.

B cell-activating factor (BAFF) and a proliferation-inducing ligand (APRIL) play crucial roles in the selection, activation, maturation, and survival of B cells. Furthermore, these cytokines are expressed in monocytes/macrophages, dendritic cells, and activated T lymphocytes [13]. Serum levels of BAFF and APRIL increase in some autoimmune diseases, such as systemic lupus erythematosus (SLE), Sjögren’s syndrome (SjS), rheumatoid arthritis, immunoglobulin G4 (IgG4)-related disease, and anti-Jo-1-positive polymyositis and dermatomyositis [14–18]. In SjS, overproduction of BAFF in the sputum has also been reported [19]. One report found that treatment of SLE with BAFF/APRIL inhibitor significantly improved the SLE responder index and safely decreased flares of severe disease relative to placebo plus standard SLE therapy [20]. In SLE model mice, serum levels of BAFF increased as a result of the disease, and BAFF blockade led to reduced disease manifestations [21]. Moreover, silencing of the BAFF gene in mice with collagen-induced arthritis abrogated the development of autoimmune arthritis [22].

Serum levels of BAFF and APRIL increase in autoimmune diseases in general [14–18], and levels of BAFF in the sputum increase in patients with SjS [19]. Furthermore, serum levels of BAFF are negatively correlated with pulmonary function test results [15]. However, there are no reports on B cell-activating factors in patients with APAP, which involves overproduction of the autoantibody against GM-CSF.

We hypothesized that BAFF and/or APRIL levels in the serum and/or bronchoalveolar lavage fluid (BALF) are elevated in this disease. Therefore, we measured the levels of both BAFF and APRIL in patients with APAP, and analyzed their association with clinical measures and the effects of treatments, in the hope of identifying novel targets for treatment.

## Materials and methods

### Study subjects

We enrolled 115 patients with APAP (mean age: 55.8 ± 13.4 years), 34 healthy volunteers (mean age: 51.4 ± 6.5 years) as controls for serum BAFF and APRIL, and 13 non-autoimmune-disease controls (mean age: 49.2 years; range: 29.7–55.5 years; median: 49.2 years) as controls for BAFF and APRIL in BALF (Table 1). The disease controls were cases with chronic obstructive pulmonary disease (n = 4), idiopathic pulmonary fibrosis (n = 2), Langerhans cell histiocytosis (n = 2), chronic bronchitis (n = 1), Castleman disease (n = 1), squamous cell carcinoma (n = 1), sporadic lymphangioleiomyomatosis (n = 1), or lymphomatoid granulomatosis (n = 1). The diagnosis of control diseases was determined via clinical, radiological, and pathological examinations. The diagnosis of APAP was confirmed based on transbronchial lung biopsy, bronchoalveolar lavage, radiological findings, clinical findings, and the presence of anti-GM-CSF autoantibody, according to the diagnostic criteria for APAP [1, 2]. The APAP DSS was evaluated, based on symptoms and PaO_2_, and rated from least (DSS 1) to most (DSS 5) severe [1].

**Table 1:** Demographic and clinical characteristics of the subjects

			APAP			Healthy Volunteer			Disease Control
Characteristics	N	%	Median (I.Q. range ) or Mean ± SD	N	%	Median (I.Q. range ) or Mean ± SD	N	%	Median (I.Q. range ) or Mean ± SD
Age – years	110		55.8 ± 13.4	34		51.4 ± 6.5	13		49.2 (29.7–55.5)
Gender									
Female	33	30		21	61.8		4	30.8	
Male	77	70		13	38.2		9	69.2	
Onset of symptom – years	110		53.0 ± 13.3						
Symptoms									
Dyspnea	82	74.5							
Cough	54	49.1							
Sputum	39	35.5							
Dust exposure									
Yes	45	40.9							
No	65	59.1							
Smoking									
Current	28	25.5		4	11.8		3	23.1	
Ex	43	39		12	35.3		5	38.5	
Never	39	35.5		16	47.1		2	15.4	
Unknown	0	0		2	5.9		3	23.1	
Pulmonary function									
VC, % predicted	98		90.3 ± 22.9				13		91.3 (79.8–111.0)
FVC, % predicted	98		88.9 ± 24.0				13		91.3 (79.7–109.1)
FEV_1.0_, % predicted	94		95.3 ± 25.0				13		79.0 (64.1–94.6)
DLco, % predicted	97		59.1 (45.6–75.9)				13		49.4 (37.6–80.3)
PaO_2_	75		68.8 ± 15.3						
Serum biomarkers									
KL-6, U/L	108		3799.5 (1601.0–8787.5)				13		328.0 (237.5–917.0)
SP-D, ng/ml	106		183.9 (120.6–311.5)				12		65.5 (39.0–134.8)
SP-A, ng/ml	81		96.7 (62.5–151.4)				13		40.1 (31.3–67.5)
LDH, IU/L	108		263.5 (217.8–339.8)						185.0 (144.0–219.5)
Anti-GM-CSF autoantibody, µg/ml	110		40.5 (17.1–71.8)						

APAP, autoimmune pulmonary alveolar proteinosis; IQR, interquartile range; SD, standard deviation; VC, vital capacity; FVC, forced expiratory volume; FEV1, forced expiratory volume in 1 s; DLco, diffusion capacity for carbon monoxide; PaO_2_, arterial partial pressure of oxygen; KL-6, Krebs von den Lungen-6; SP-D, surfactant protein-D; SP-A, surfactant protein-A; LDH; lactate dehydrogenase; GM-CSF, granulocyte-macrophage colony-stimulating factor. Data are expressed as the median (IQR) or mean ± SD

This study was approved by the ethics committee of the National Hospital Organization Kinki-Chuo Chest Medical Center (Sakai, Japan) (approval numbers: 365 and 576). Written informed consent was provided by all subjects.

### Measurement of BAFF and APRIL in the serum and BALF

All serum samples were collected and stored at − 80 ˚C until examination.

The levels of BAFF in the serum and BALF were measured using an enzyme-linked immunosorbent assay (ELISA) kit according to the instructions provided by the manufacturer (R&D Systems, Minneapolis, MN, USA). The levels of APRIL in the serum and BALF were measured with an ELISA kit according to the instructions provided by the manufacturer (eBioscience, Vienna, Austria). These assays were performed in duplicate.

### Serum biomarkers

The levels of Krebs von den Lungen-6 (KL-6) (Picolumi KL-6; EIDIA, Tokyo, Japan), SP-D (SP-D kit YAMASA EIA II; Kyowa Medex, Chiba, Japan), SP-A (SP-A test Kokusai-F kit; SYSMEX, Kobe, Japan), and lactate dehydrogenase (LDH) (Cica Liquid LDH J; Kanto Chemical, Tokyo, Japan) in the serum were measured. The reference levels of normal human serum in this study were KL-6 (< 500 U/mL), SP-D (< 110 ng/mL), SP-A (< 48.3 ng/mL), and LDH (115 < LDH < 229 IU/L).

We measured the levels of anti-GM-CSF autoantibody in the serum using a specific previously reported ELISA method [1, 23, 24] with partial modification.

### Pulmonary function testing

Certified pulmonary function technicians performed pulmonary function tests using a CHESTAC-8800 (Chest M.I., Tokyo, Japan). The data collected included the percentages of vital capacity (VC), forced vital capacity (FVC), forced expiratory volume in 1 s (FEV1), and single-breath diffusion capacity for carbon monoxide (DLco). Each pulmonary function measurement was expressed as a percent predicted value. Acceptability and reproducibility criteria from the American Thoracic Society’s recommendations for standardization were used to assess the validity of each testing session [25].

### WLL treatment

After induction of general anesthesia, patients were intubated, while supine, with a left-side double-lumen endotracheal tube and their lungs were ventilated mechanically with 100 % oxygen. The lung was lavaged with warmed (37°C) normal saline using aliquots of 500 mL at a pressure of 30 cmH_2_O. The lavage was terminated when the color of the lavage fluid changed from milky to clear; the total volume of saline delivered to a single lung was typically 15–20 L [26]. Recovered WLL fluid was filtered with a gauze filter and centrifuged at 44 × g for 5 min, and the supernatant was stored at − 30°C until use.

### Bronchoalveolar lavage

Bronchoalveolar lavage was performed as previously described [27] via flexible bronchoscopy. Aliquots of sterile saline (50 mL) at 37°C were injected thrice through the bronchoscope for clinical usage and gently aspirated with a syringe 4th BALF for this study. The BALF was filtered using a gauze filter and centrifuged at 44 × g for 5 min, and the supernatant was stored at − 30°C until use.

### Treatment with inhalation of recombinant human GM-CSF

Two patients received rhGM-CSF (125 µg in 2 mL of sterile saline; Molgramostim, Leucomax; Novartis AG, Basel, Switzerland) via inhalation of aerosol using an LC-PLUS nebulizer with a manual interrupter valve connected to a PARI Turbo BOY compressor (PARI, Starnberg, Germany). The treatment was administered twice daily, every second week, for 24 weeks [28]. Fifteen patients received rhGM-CSF (125 µg in 2 mL of sterile saline; Sargramostim, Leukine lyophilized formulation; Berlex, Seattle, WA, USA) via inhalation of aerosol using an LC-PLUS nebulizer with a manual interrupter valve connected to a PARI Turbo BOY compressor [10]. Treatments included the two effective regimens of varying rhGM-CSF dosage [9, 28]. Clinical response was defined as a reduction in alveolar–arterial O_2_ tension difference of at least 10 torr at the end of treatment relative to baseline [9].

### Statistical analysis

Statistical analyses were performed using the JMP software, Version 10.0 (SAS Institute, Cary, NC, USA). Numerical variables are expressed as median (interquartile range) and were assessed using the Wilcoxon rank-sum test. Correlations of variables were assessed using Spearman’s rank correlation coefficient. Steel’s test was used to compare the levels of BAFF and APRIL before rhGM-CSF inhalation and WLL with after treatment. Differences were considered statistically significant at p < 0.05.

## Results

### Baseline levels of BAFF and APRIL in serum and BALF

**Fig. 1:**
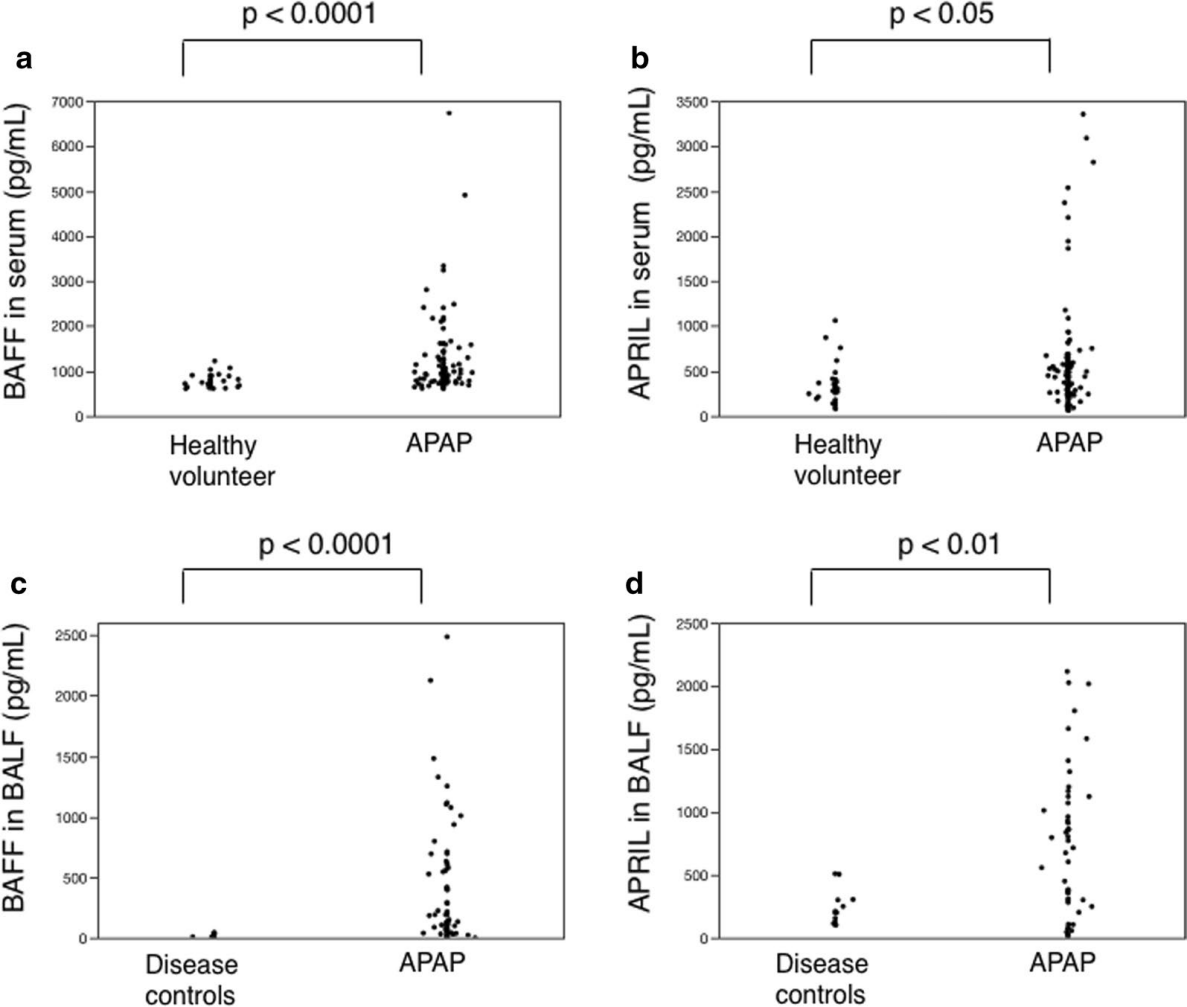
Levels of BAFF and APRIL in 115 patients with APAP. Serum levels of BAFF (**a**) and APRIL (**b**) were significantly elevated in patients with APAP relative to healthy volunteers (p < 0.0001 and p < 0.05, respectively). Levels of BAFF (**c**) and APRIL (**d**) in BALF were significantly elevated in patients with APAP versus disease controls (p < 0.0001 and p < 0.01, respectively).  APAP, autoimmune pulmonary alveolar proteinosis; APRIL, a proliferation-inducing ligand; BAFF, B cell-activating factor; BALF, bronchial alveolar lavage fluid

The levels of BAFF and APRIL in the serum of patients with APAP were significantly higher than those in healthy volunteers (p < 0.0001 and p < 0.05, respectively; Fig. 1a, b). Median levels of BAFF in the serum were 904.8 (780.5–1,252.8) pg/mL in patients with APAP and 735.5 (652.2–891.0) pg/mL in healthy volunteers. Median levels of APRIL in the serum were 435.4 (243.3–586.5) pg/mL in patients with APAP and 303.4 (203.1–401.9) pg/mL in healthy volunteers.

The levels of BAFF and APRIL in the BALF of patients with APAP were significantly higher than those in disease controls (p < 0.0001 and p < 0.01, respectively; Fig. 1c, d). Median levels of BAFF in the BALF were 191.4 (88.4–703.3) pg/mL in patients with APAP and 8.1 (4.85–26.9) pg/mL in disease controls. Median levels of APRIL in the BALF were 639.5 (237.5–1,026.8) pg/mL in patients with APAP and 201.0 (118.5–303.0) pg/mL in disease controls. There were two cases that showed a marked increase in serum levels of BAFF (Fig. 1). Of these, one had BAFF levels of 6737.5 pg/mL and was complicated by non-tuberculous mycobacteriosis, and serum anti GM-CSF autoantibody level was 36.9 µg/ml. The other had the BAFF level of 4910.4 pg/mL, exhibited a marked increase I KL-6 (70,010 U/mL), carcinoembryonic antigen (80.0 ng/mL), cytokeratin subunit 19 fragment (53.0 ng/mL), and was complicated by myelodysplastic syndrome (MDS). Serum anti-GM-CSF autoantibody was 21.4 µg/mL. Pathological and cytological findings of PAP were confirmed about these two patients, and our final diagnosis were APAP (not secondary PAP) complicated with NTM or MDS from the definition of APAP.

Levels of both BAFF and APRIL in the serum and BALF were correlated (p = 0.009 and p = 0.001, respectively). However, levels of BAFF in the serum and APRIL in the BALF were negatively correlated (p = 0.005; Table 2).

**Table 2:** Association between B cell-activating factors in the serum and BALF

	BAFF in serum	APRIL in serum	BAFF in BALF	APRIL in BALF
BAFF in serum		0.14 (0.14)	0.33 (0.009*)	− 0.40 (0.004*)
APRIL in serum			0.09 (0.51)	0.36 (0.001*)
BAFF in BALF				− 0.27 (0.06)
APRIL in BALF				

BAFF, B cell-activating factor; APRIL, a proliferation-inducing ligand; BALF, bronchoalveolar lavage fluid. Data were analyzed using Spearman’s correlation coefficient. *Significant at the 0.05 level

The baseline levels of BAFF and APRIL in the serum and BALF were not correlated with dyspnea, cough, sputum, smoking, or exposure to dust (Table 3). The levels of BAFF in the serum and BALF were significantly correlated with DSS (p = 0.04 and p = 0.007, respectively). However, levels of APRIL in the serum and BALF were not correlated with DSS (Table 3).

**Table 3:** Correlations between symptoms and baseline levels of BAFF and APRIL

	**BAFF**		**APRIL**	
Symptom	Serum	BALF	Serum	BALF
Dyspnea	0.15	0.08	0.38	0.18
Cough	0.80	0.98	0.81	0.72
Sputum	0.40	0.97	0.54	0.67
Smoking	0.30	0.18	0.33	0.26
Exposure to dust	0.79	0.32	0.65	0.31
DSS	0.04*	0.007*	0.25	0.90

BAFF, B cell-activating factor; APRIL, a proliferation-inducing ligand; DSS, disease severity score. Data except DSS were analyzed using Wilcoxon rank-sum test. Data of DSS were analyzed using Spearman’s correlation coefficient. *Significant at the 0.05 level

### Correlations between BAFF, APRIL, and serum biomarkers at baseline

We confirmed the previously reported elevation of serum biomarkers for APAP in patients with APAP [1, 28, 29]. The levels of KL-6, SP-D, SP-A, and LDH in the serum were significantly correlated with those of BAFF (p < 0.0001). However, only the levels of APRIL in the serum were correlated with SP-D (p = 0.04). Serum levels of BAFF and APRIL were not correlated with anti-GM-CSF autoantibody (Table 4). Among the serum biomarkers examined in this study, only SP-A showed correlation with anti-GM-CSF autoantibody (data not shown).

**Table 4:** Correlations between serum biomarkers and baseline levels of BAFF and APRIL

Biomarker	BAFF		APRIL	
	ρ	p	ρ	p
KL-6	0.50	< 0.0001*	0.15	0.12
SP-D	0.48	< 0.0001*	0.20	0.04*
SP-A	0.43	< 0.0001*	0.15	0.17
LDH	0.47	< 0.0001*	0.13	0.17
GM-CSF autoantibody	0.13	0.19	0.07	0.14

Abbreviations: BAFF, B cell-activating factor; APRIL, a proliferation-inducing ligand; KL-6, Krebs von den Lungen-6; SP-D, surfactant protein-D; SP-A, surfactant protein-A; LDH; lactate dehydrogenase; GM-CSF, granulocyte-macrophage colony-stimulating factor. Data were analyzed using Spearman’s correlation coefficient. *Significant at the 0.05 level

### Correlations between BAFF and APRIL levels and pulmonary functions at baseline

We evaluated the relationship between the levels of BAFF and APRIL (in the serum and BALF) and pulmonary functions at baseline. Serum levels of BAFF were significantly negatively correlated with % predicted DLco (p = 0.004), but were not correlated with % predicted VC, FVC, or FEV1 (Table 5). Serum levels of APRIL were significantly negatively correlated with % predicted FVC (p = 0.04), but were not significantly correlated with % predicted VC, FEV1, or DLco (Table 5).

**Table 5:** Correlations between pulmonary functions and baseline levels of BAFF and APRIL in serum

Pulmonary function	BAFF in serum		APRIL in serum	
	ρ	p	ρ	p
VC (% predicted)	− 0.17	0.10	− 0.15	0.15
FVC (% predicted)	− 0.14	0.17	− 0.21	0.04*
FEV1 (% predicted)	− 0.08	0.46	− 0.04	0.71
DLco (% predicted)	− 0.29	0.004*	− 0.15	0.15

BAFF, B cell-activating factor; APRIL, a proliferation-inducing ligand; VC, vital capacity; FVC, forced expiratory volume; FEV1, forced expiratory volume in 1 s; DLco, diffusion capacity for carbon monoxide. Data were analyzed using Spearman’s correlation coefficient. *Significant at the 0.05 level

The levels of BAFF in the BALF were significantly negatively correlated with % predicted VC (p = 0.04) and DLco (p = 0.006), but there were no significant correlations between APRIL in the BALF and the pulmonary functions (Table 6).

**Table 6:** Correlations between pulmonary functions and levels of BAFF and APRIL in BALF

	**BAFF in BALF**		**APRIL in BALF**	
Pulmonary function	ρ	p	ρ	p
VC (% predicted)	−0.29	0.04*	−0.22	0.14
FVC (% predicted)	−0.24	0.07	−0.25	0.10
FEV1 (% predicted)	−0.08	0.59	−0.002	0.99
DLco (% predicted)	−0.34	0.006*	−0.05	0.77

BAFF, B cell-activating factor; APRIL, a proliferation-inducing ligand; BALF, bronchoalveolar lavage fluid; VC, vital capacity; FVC, forced expiratory volume; FEV1, forced expiratory volume in 1 s; DLco, diffusion capacity for carbon monoxide. Data were analyzed using Spearman’s correlation coefficient. *Significant at the 0.05 level

### Effects of treatment on serum levels of BAFF and APRIL

**Fig. 2:**
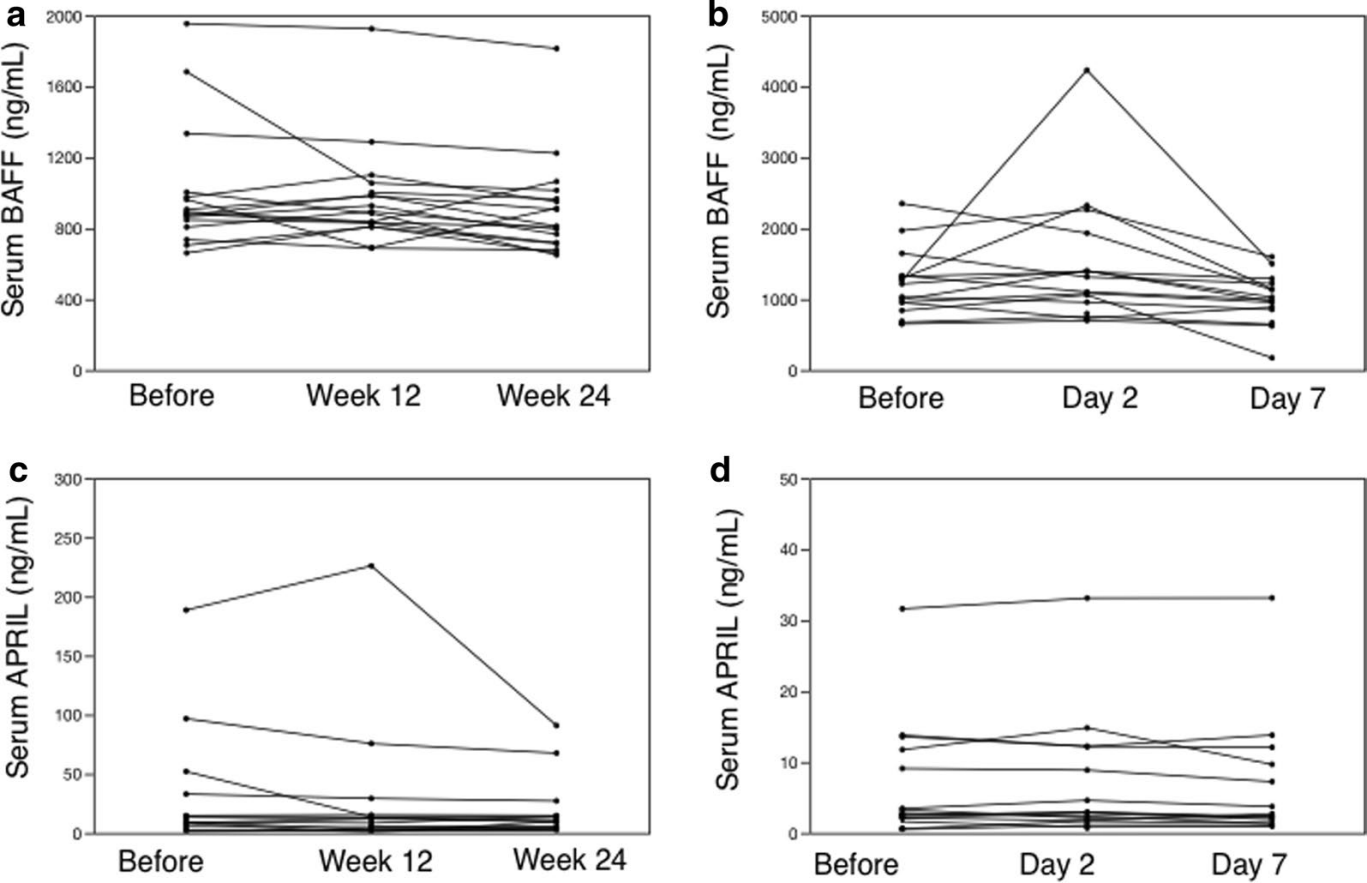
Change in serum levels of BAFF and APRIL before and after treatment with **a** rhGM-CSF inhalation therapy (N = 17) and **b** whole lung lavage (N = 16). Change in serum levels of APRIL before and after treatment with **c** rhGM-CSF inhalation (N = 17) and **d** whole lung lavage (N = 16). Abbreviations: BAFF, B cell-activating factor; APRIL, a proliferation-inducing ligand; GM-CSF, granulocyte-macrophage colony-stimulating factor. Data were analyzed using the Steel’s test

Seventeen patients with APAP were treated with rhGM-CSF. We evaluated their serum BAFF and APRIL levels at baseline and after 12 and 24 weeks of treatment, but did not detect any significant treatment effects (Fig. 2a and c) same as anti-GM-CSF autoantibody (data not shown). However, we confirmed the improvement of serum level of KL-6 and DSS with rhGM-CSF (data not shown) as in the previous report [12, 28]. In order to assess the effects of rhGM-CSF inhalation on BAFF and APRIL, the patients were treated with inhaled GM-CSF under the protocols previously reported [9, 28]. In fact, serum levels of BAFF and APRIL were unaffected regardless of response to rhGM-CSF inhalation therapy and despite differences in history of smoking and dust exposure (data not shown).

WLL was performed in 16 patients with APAP, and we evaluated their serum levels of BAFF and APRIL at baseline and two and seven days after treatment. Similarly to our findings for GM-CSF inhalation therapy, we did not detect a significant effect of the treatment on the serum levels of BAFF or APRIL (Fig. 2b, d).

## Discussion

This is the first report to demonstrate the overproduction of the B cell-activating factors BAFF and APRIL in patients with APAP relative to healthy volunteers (in the serum) and disease controls (in the BALF) (Fig. 1). Furthermore, the levels of BAFF in both serum and BALF were correlated with DSS (Table 3), and the serum levels were correlated with serum biomarkers for APAP (Table 4). However, the treatments for APAP (i.e., inhalation of rhGM-CSF and WLL) did not affect serum levels of BAFF or APRIL (Fig. 2), or those of the anti-GM-CSF autoantibody.

### Baseline levels of BAFF and APRIL in the serum and BALF

As predicted by our hypothesis, serum levels of BAFF and APRIL were elevated in patients with APAP, as in other autoimmune diseases [14, 16–18]. However, median serum levels of BAFF in patients with APAP were approximately 32 %, 40 %, and 15 % of those reported in SLE [30], IgG4-related disease [14], and SjS [31], respectively, and those of APRIL were approximately 5 %, 20 %, and 20 % of those in SLE [30], IgG4-related disease [14], and SjS [32], respectively. This suggests that autoimmunity such as production of autoantibody in APAP may be lower than in these three other autoimmune diseases.

Two cases showed a marked increase in serum BAFF levels as indicated above (Fig. 1). In one of these, the patient’s condition was complicated by non-tuberculous mycobacteriosis. One study has shown that the dramatic elevation of BAFF and APRIL levels in the plasma and pleural effusion of patients with tuberculous pleurisy, and the BAFF/APRIL system itself, was closely related to the T helper 1 (Th1) immune response [33]. The enhancement of BAFF and APRIL in APAP, an organ-specific autoimmune disease, suggests an association between its pathogenesis and the Th1 immune response. In the other case, the case was complicated by MDS. The pathogenic mechanism of MDS might affect serum BAFF level via functional and qualitative abnormalities of T cell, B cell, and Natural killer cell, but the clear mechanism remains unclear [34, 35].

Although clinical symptoms were not correlated with BAFF and APRIL in the serum or BALF, BAFF levels in both were correlated with DSS (Table 3). This suggests that BAFF (rather than APRIL) may be important for disease progression. In the circulation system, BAFF occurs in soluble trimmer form and APRIL is present in both soluble trimmer form and as a multimer form in association with heparin sulfate proteoglycans. In addition, there are three types of receptors for BAFF and APRIL, which exhibit different binding abilities [13]. Measurement of BAFF may facilitate noninvasive determination of DSS without arterial blood sampling. The correlation of the levels of BAFF in the serum and BALF (Table 2) were similar to results previously reported for patients with sarcoidosis [36]. These results suggest that BAFF may be more closely associated than APRIL with the pathogenesis of APAP.

### Correlations between BAFF, APRIL, and serum biomarkers at baseline

We confirmed the high concentrations of serum biomarkers that are useful for APAP diagnosis and disease evaluation [1, 28, 29]. In addition, these serum biomarkers were correlated with serum levels of BAFF, but not APRIL (Table 4). Based on experimental data regarding B cell depletion, both of these cytokines release signals to promote the differentiation and longevity of B cells, although certain immune-modulating aspects may differ [30]. The different binding abilities of the receptors for BAFF and APRIL [13] may affect the etiology and progression of APAP.

We confirmed that the levels of anti-GM-CSF autoantibody did not reflect the DSS, as previously reported for APAP [37, 38]. However, we did find that BAFF was correlated with the DSS (Table 4). Since BAFF is a B cell-activation factor, this suggests that B cell activation affects the pathogenesis of APAP. BAFF exhibits similar behavior to that of the serum biomarkers for APAP, and may thus be a promising biomarker for disease progression.

### Correlations between BAFF and APRIL levels and pulmonary functions at baseline

The baseline levels of BAFF in the serum and BALF were negatively correlated with baseline DLco (Tables 5, 6). The concentration of BAFF is high in cases that are so severe that gas exchange problems occur, so the correlation between BAFF levels and DSS makes sense (Table 3). The age of the APAP patients participating in this study may also have exacerbated their disease severity, since it is an extrapulmonary factor connected with decreasing diffusion capacity.

### Effects of treatment on serum levels of BAFF and APRIL

In our study, serum levels of BAFF and APRIL did not change significantly after WLL or rhGM-CSF inhalation therapy (Fig. 2). Although BAFF and APRIL present locally in the lungs are temporarily removed by WLL, they are soon replenished because the treatment does not remove the cells that produce them [39]. In contrast, it is conceivable that rhGM-CSF inhalation therapy exerts a negative effect on BAFF and APRIL levels, because the alveolar macrophages activated by rhGM-CSF treatment are one of the types of cells that produce BAFF and APRIL. The reason for the ineffectiveness of the current APAP treatments in reducing BAFF and APRIL levels may be that these treatments do not act directly on the concentrations of these cytokines. Interestingly, there were some cases with decreased levels of BAFF and APRIL after treatment. However, these differences were not dependent on a history of smoking [1] or dust exposure [40], which are considered to be risk factors for APAP (data not shown). There may also be other reasons why the treatments did not directly affect the levels of BAFF, APRIL, or the anti-GM-CSF autoantibody in APAP. Neutrophils producing BAFF maintain autoantibody production in settings of autoimmunity and cancer [41]. Hence, it has been suggested that the use of medicine that suppresses B cell-activating factors, such as belimumab [42], may be effective for the treatment of APAP. There were reports about the effectiveness of anti-CD20 antibody (rituximab) treatment on pulmonary functions [43] and on alveolar macrophage lipid metabolism by increasing lipid transport and surface catabolism in autoimmune pulmonary alveolar proteinosis. Suggested mechanisms of these effects are GM-CSF stimulation of alveolar macrophage ATP-binding cassette transporter lipid homeostasis and Lysosomal phospholipase A2 [44]. CD20 is widely expressed from naïve B cell to memory B cell. However, rituximab does not affect plasma cells which lack CD20 expression. Since the BAFF receptor is also expressed in plasma cells [45, 46], a therapy targeting BAFF could be effective on plasma cells too. In our study, inhaled rhGM-CSF and WLL could not attenuate serum levels of BAFF and APRIL in APAP. There are several reports that serum BAFF levels increased after the treatment of anti-CD20 antibody [45] in patients with autoimmune diseases.

Suppression of B cell-activating factors could be a new candidate of future APAP treatment as suggested in autoimmune diseases [45]. Further multicenter longitudinal studies are warranted to further investigate the role of BAFF and APRIL and therapeutic target in APAP.

### Limitations

There were several limitations in this study, including the retrospective nature of the investigation, the single-center cohort, and the small number of patients with non-autoimmune diseases.

## Conclusions

BAFF and APRIL levels of sera and BALF in APAP were significantly increased compared with healthy volunteer and disease control, and the BAFF and APRIL pathway might have important specific roles in pathogenesis of APAP. Our data suggest a new perspective of future treatment for APAP. 

## Data Availability

The datasets generated and analyzed during the current study are not publicly available, to ensure the anonymity of the subjects. The datasets are available from the corresponding author upon reasonable request.

## Abbreviations

APAP: Autoimmune pulmonary alveolar proteinosis
GM-CSF: Granulocyte-macrophage colony-stimulating factor
BAFF: B cell-activating factor
APRIL: A proliferation-inducing ligand
WLL: Whole lung lavage
BALF: Bronchoalveolar lavage fluid
DSS: Disease severity score
SLE: Systemic lupus erythematosus
SjS: Sjögren’s syndrome
KL-6: Krebs von den Lungen-6;
SP: Surfactant protein
LDH: Lactate dehydrogenase
ELISA: Enzyme-linked immunosorbent assay
VC: Vital capacity
FVC: Forced vital capacity
FEV1: Forced expiratory volume in one second
DLco: Single-breath diffusion capacity for carbon monoxide;
Th: T helper
